# (*E*)-1-(2-Fur­yl)-3-(3,4,5-trimeth­oxy­phen­yl)prop-2-en-1-one

**DOI:** 10.1107/S160053681004451X

**Published:** 2010-11-06

**Authors:** Hoong-Kun Fun, Thitipone Suwunwong, Suchada Chantrapromma, Chatchanok Karalai

**Affiliations:** a X-ray Crystallography Unit, School of Physics, Universiti Sains Malaysia, 11800 USM, Penang, Malaysia; b Crystal Materials Research Unit, Department of Chemistry, Faculty of Science, Prince of Songkla University, Hat-Yai, Songkhla 90112, Thailand

## Abstract

The title mol­ecule, C_16_H_16_O_5_, is twisted; the dihedral angle between the furan and 3,4,5-trimeth­oxy­phenyl rings is 12.14 (13)°. The two meth­oxy groups at the *meta* positions of the benzene ring are close to being coplanar with the ring [C—O—C—C = −0.6 (3) and 1.4 (3)°], whereas the third meth­oxy group, at the *para* position, is (+)-anti­clinal with respect to the benzene ring [C—O—C—C = 104.9 (2)°]. In the crystal, mol­ecules are linked by weak C—H⋯O bonds to stack along the *b* axis and further C—H⋯O inter­actions consolidate the structure.

## Related literature

For bond-length data, see: Allen *et al.* (1987 ▶). For hydrogen bond motifs, see: Bernstein *et al.* (1995 ▶). For related structures, see: Fun *et al.* (2010 ▶); Suwunwong *et al.* (2009 ▶). For background to and applications of chalcones, see: Batovska *et al.* (2007 ▶); Gu *et al.* (2009 ▶); Jung *et al.* (2008 ▶); Prasad *et al.* (2008 ▶); Saxena *et al.* (2007 ▶); Tewtrakul *et al.* (2003 ▶).
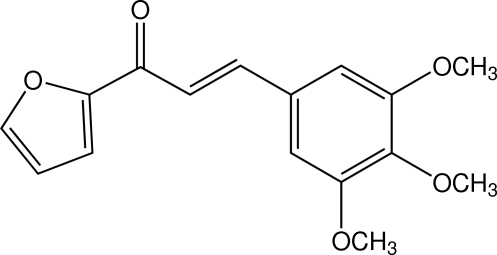


## Experimental

### 

#### Crystal data

C_16_H_16_O_5_
*M* *_r_* = 288.29
Orthorhombic, 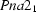

*a* = 24.2677 (4) Å
*b* = 3.9916 (1) Å
*c* = 14.0816 (2) Å
*V* = 1364.04 (5) Å^3^
*Z* = 4
Mo *K*α radiation
μ = 0.11 mm^−1^
*T* = 100 K
0.35 × 0.24 × 0.21 mm

#### Data collection

Bruker APEXII CCD diffractometer
Absorption correction: multi-scan (*SADABS*; Bruker, 2005 ▶) *T*_min_ = 0.965, *T*_max_ = 0.978
17261 measured reflections
2063 independent reflections
1907 reflections with *I* > 2σ(*I*)
*R*_int_ = 0.035

#### Refinement

*R*[*F* ^2^ > 2σ(*F* ^2^)] = 0.038
*wR*(*F* ^2^) = 0.092
*S* = 1.06
2063 reflections
254 parameters
1 restraint
All H-atom parameters refined
Δρ_max_ = 0.36 e Å^−3^
Δρ_min_ = −0.22 e Å^−3^

### 

Data collection: *APEX2* (Bruker, 2005 ▶); cell refinement: *SAINT* (Bruker, 2005 ▶); data reduction: *SAINT*; program(s) used to solve structure: *SHELXTL* (Sheldrick, 2008 ▶); program(s) used to refine structure: *SHELXTL*; molecular graphics: *SHELXTL*; software used to prepare material for publication: *SHELXTL* and *PLATON* (Spek, 2009 ▶).

## Supplementary Material

Crystal structure: contains datablocks global, I. DOI: 10.1107/S160053681004451X/hb5713sup1.cif

Structure factors: contains datablocks I. DOI: 10.1107/S160053681004451X/hb5713Isup2.hkl

Additional supplementary materials:  crystallographic information; 3D view; checkCIF report

## Figures and Tables

**Table 1:** Hydrogen-bond geometry (Å, °)

*D*—H⋯*A*	*D*—H	H⋯*A*	*D*⋯*A*	*D*—H⋯*A*
C14—H14*B*⋯O1^i^	0.99 (3)	2.54 (3)	3.503 (3)	166 (2)
C15—H15*B*⋯O4^ii^	1.01 (3)	2.36 (3)	3.337 (3)	162 (2)

Symmetry codes: (i) 
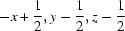
; (ii) 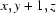.

**Fig. 1.**
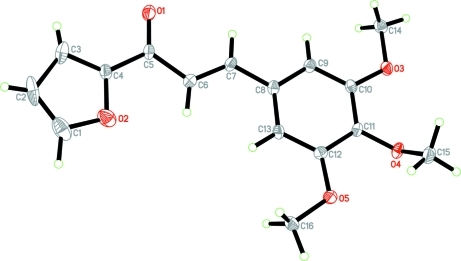
The molecular structure of (III), showing 50% probability displacement ellipsoids.

**Fig. 2.**
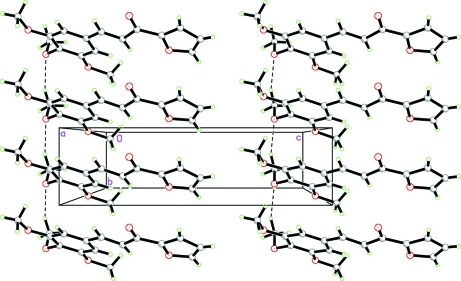
The crystal packing of (III), showing column along the b axis. C—H···O weak interactions are shown as dashed lines.

## supplementary crystallographic information

### Comment

Chalcones are known to exhibit bioactivity including antimicrobial (Prasad *et al.*, 2008), antifungal (Batovska *et al.*, 2007), anticancer (Saxena *et al.*, 2007) and HIV-1 protease inhibitory (Tewtrakul *et al.*, 2003), as well as Non Linear Optical (NLO) (Gu *et al.*, 2009) and fluorescence properties (Jung *et al.*, 2008). In our on-going research on antibacterial activities, NLO and fluorescence properties of aryl/heteroaryl chalcones, we previously reported the crystal structures of (*E*)-1-(2-furyl)-3-(2,4,6-trimethoxyphenyl)prop-2-en-1-one (I) (Fun *et al.*, 2010) and (*E*)-1-(2-thienyl)-3-(3,4,5-trimethoxyphenyl)prop-2-en-1-one (II) (Suwunwong *et al.*, 2009). The title compound (III) was synthesized and its crystal structure was determined in order to gain structural details to explain the affects of the substituent groups and their positions on the fluorescence properties and how their crystal packing would affect the NLO properties of the compounds. Compound (I) crystallized out in the centrosymmetric *C2/c* space group which prohibits second order NLO properties whereas compounds (II) and (III) crystallized in non-centrosymmetric *Pna*2_1_ space group and should possess the second order NLO properties.

The molecule of the title heteroaryl chalcone (Fig. 1) exists in an *E* configuration with respect to the C6═C7 double bond [1.340 (3) Å] with torsion angle C5–C6–C7–C8 = 174.83 (19)°. The molecule is twisted with the dihedral angle between the furan and 3,4,5-trimethoxyphenyl rings being 12.14 (13)°. The propenone unit (C5—C7/O1) is slightly deviated with the torsion angle O1–C5–C6–C7 = 8.0 (3)°. The three methoxy groups of the 3,4,5-trimethoxyphenyl unit have two different orientations: the two methoxy groups at the *meta* positions (at atom C10 and C12 positions) are co-planar with the attached benzene ring with torsion angles C14–O3–C10–C9 = -0.6 (3)° and C16–O5–C12–C13 = 1.4 (3)° whereas the third one at *para* position (at atom C11) is (+)-anti-clinally attached with the torsion angle C15–O4–C11—C10 = 104.9 (2)°. Otherwise, the bond distances in (III) are of normal values (Allen *et al.*, 1987) and are comparable with the closely related structures (Fun *et al.*, 2010; Suwunwong *et al.*, 2009).

In the crystal (Fig. 2), the molecules are stacked into columns along the *b* axis and molecules within the stacks are linked by weak C15—H15B···O4 (Table 1) interactions.

### Experimental

The title compound was synthesized by the condensation of 3,4,5-trimethoxybenzaldehyde (0.39 g, 2 mmol) in ethanol (15 ml) with 2-furyl methylketone (0.22 g, 2 mmol) in ethanol (15 ml) in the presence of 20% NaOH(aq) (5 ml). After stirring for 4 h, the resulting pale yellow solid appeared and was then collected by filtration, washed with distilled water and dried (69% yield). Pale yellow blocks of (III) were recrystalized from acetone/methanol (1:1 *v*/*v*) by the slow evaporation of the solvent at room temperature after several days, Mp. 420–421 K.

### Refinement

All H atoms were located in difference maps and refined isotropically. The highest residual electron density peak is located at 0.89 Å from C2 and the deepest hole is located at 0.70 Å from O2. A total of 1892 Friedel pairs were merged before final refinement.

### Crystal data

**Table:**

C_16_H_16_O_5_	*D*_x_ = 1.404 Mg m^−^^3^
*M**_r_* = 288.29	Melting point = 420–421 K
Orthorhombic, *P**n**a*2_1_	Mo *K*α radiation, λ = 0.71073 Å
Hall symbol: P 2c -2n	Cell parameters from 2063 reflections
*a* = 24.2677 (4) Å	θ = 2.2–30.0°
*b* = 3.9916 (1) Å	µ = 0.11 mm^−^^1^
*c* = 14.0816 (2) Å	*T* = 100 K
*V* = 1364.04 (5) Å^3^	Block, pale yellow
*Z* = 4	0.35 × 0.24 × 0.21 mm
*F*(000) = 608	

### Data collection

**Table:**

Bruker APEXII CCD diffractometer	2063 independent reflections
Radiation source: sealed tube	1907 reflections with *I* > 2σ(*I*)
graphite	*R*_int_ = 0.035
φ and ω scans	θ_max_ = 30.0°, θ_min_ = 2.2°
Absorption correction: multi-scan (*SADABS*; Bruker, 2005)	*h* = −29→34
*T*_min_ = 0.965, *T*_max_ = 0.978	*k* = −5→5
17261 measured reflections	*l* = −19→19

### Refinement

**Table:**

Refinement on *F*^2^	Primary atom site location: structure-invariant direct methods
Least-squares matrix: full	Secondary atom site location: difference Fourier map
*R*[*F*^2^ > 2σ(*F*^2^)] = 0.038	Hydrogen site location: inferred from neighbouring sites
*wR*(*F*^2^) = 0.092	All H-atom parameters refined
*S* = 1.06	*w* = 1/[σ^2^(*F*_o_^2^) + (0.0501*P*)^2^ + 0.4265*P*] where *P* = (*F*_o_^2^ + 2*F*_c_^2^)/3
2063 reflections	(Δ/σ)_max_ = 0.001
254 parameters	Δρ_max_ = 0.36 e Å^−^^3^
1 restraint	Δρ_min_ = −0.22 e Å^−^^3^

### Special details

**Table:**

Experimental. The crystal was placed in the cold stream of an Oxford Cryosystems Cobra open-flow nitrogen cryostat (Cosier & Glazer, 1986) operating at 120.0 (1) K.
Geometry. All e.s.d.'s (except the e.s.d. in the dihedral angle between two l.s. planes) are estimated using the full covariance matrix. The cell e.s.d.'s are taken into account individually in the estimation of e.s.d.'s in distances, angles and torsion angles; correlations between e.s.d.'s in cell parameters are only used when they are defined by crystal symmetry. An approximate (isotropic) treatment of cell e.s.d.'s is used for estimating e.s.d.'s involving l.s. planes.
Refinement. Refinement of *F*^2^ against ALL reflections. The weighted *R*-factor *wR* and goodness of fit *S* are based on *F*^2^, conventional *R*-factors *R* are based on *F*, with *F* set to zero for negative *F*^2^. The threshold expression of *F*^2^ > σ(*F*^2^) is used only for calculating *R*-factors(gt) *etc*. and is not relevant to the choice of reflections for refinement. *R*-factors based on *F*^2^ are statistically about twice as large as those based on *F*, and *R*- factors based on ALL data will be even larger.

### Fractional atomic coordinates and isotropic or equivalent isotropic displacement parameters (Å^2^)

**Table:**

	*x*	*y*	*z*	*U*_iso_*/*U*_eq_	
O1	0.25005 (6)	0.6063 (4)	0.72174 (11)	0.0251 (3)	
O2	0.34121 (7)	0.1635 (4)	0.87377 (12)	0.0271 (4)	
O3	0.41052 (6)	0.5004 (4)	0.26993 (10)	0.0181 (3)	
O4	0.50168 (6)	0.1761 (4)	0.32189 (11)	0.0181 (3)	
O5	0.51882 (6)	−0.0146 (4)	0.50322 (11)	0.0190 (3)	
C1	0.33965 (11)	0.1379 (8)	0.96954 (19)	0.0333 (6)	
H1A	0.3722 (14)	0.001 (7)	0.995 (2)	0.036 (8)*	
C2	0.29645 (12)	0.3011 (8)	1.00503 (17)	0.0342 (6)	
H2A	0.2846 (17)	0.331 (11)	1.057 (3)	0.064 (13)*	
C3	0.26677 (10)	0.4471 (7)	0.92549 (17)	0.0247 (5)	
H3A	0.2426 (12)	0.568 (8)	0.922 (2)	0.026 (8)*	
C4	0.29621 (8)	0.3538 (5)	0.84793 (14)	0.0171 (4)	
C5	0.28952 (8)	0.4380 (5)	0.74728 (14)	0.0168 (4)	
C6	0.33353 (8)	0.3224 (5)	0.68267 (15)	0.0171 (4)	
H6A	0.3628 (11)	0.190 (7)	0.709 (2)	0.020 (6)*	
C7	0.33640 (8)	0.4294 (5)	0.59260 (14)	0.0167 (4)	
H7A	0.3082 (12)	0.566 (7)	0.570 (2)	0.023 (7)*	
C8	0.37985 (8)	0.3565 (5)	0.52384 (13)	0.0155 (4)	
C9	0.37237 (8)	0.4630 (5)	0.43008 (15)	0.0154 (3)	
H9A	0.3401 (11)	0.568 (7)	0.410 (2)	0.019 (6)*	
C10	0.41374 (8)	0.4064 (5)	0.36290 (13)	0.0143 (3)	
C11	0.46246 (8)	0.2434 (5)	0.38936 (13)	0.0145 (3)	
C12	0.46971 (8)	0.1383 (5)	0.48394 (14)	0.0151 (4)	
C13	0.42857 (8)	0.1923 (5)	0.55057 (14)	0.0155 (4)	
H13A	0.4332 (10)	0.125 (6)	0.615 (2)	0.014 (6)*	
C14	0.36071 (8)	0.6651 (6)	0.24098 (15)	0.0189 (4)	
H14C	0.3554 (11)	0.873 (7)	0.274 (2)	0.020 (7)*	
H14B	0.3290 (12)	0.513 (7)	0.248 (2)	0.019 (7)*	
H14A	0.3628 (12)	0.717 (7)	0.177 (2)	0.023 (7)*	
C15	0.54913 (9)	0.3915 (6)	0.32550 (18)	0.0224 (4)	
H15A	0.5639 (17)	0.395 (12)	0.393 (3)	0.069 (13)*	
H15B	0.5367 (14)	0.627 (8)	0.309 (2)	0.040 (9)*	
H15C	0.5741 (12)	0.315 (8)	0.277 (2)	0.032 (8)*	
C16	0.52677 (9)	−0.1308 (6)	0.59880 (14)	0.0198 (4)	
H16A	0.4975 (11)	−0.287 (7)	0.617 (2)	0.022 (7)*	
H16B	0.5618 (12)	−0.249 (7)	0.598 (2)	0.023 (7)*	
H16C	0.5293 (11)	0.058 (8)	0.645 (2)	0.021 (7)*	

### Atomic displacement parameters (Å^2^)

**Table:**

	*U*^11^	*U*^22^	*U*^33^	*U*^12^	*U*^13^	*U*^23^
O1	0.0218 (7)	0.0366 (8)	0.0169 (7)	0.0091 (7)	0.0035 (6)	0.0052 (7)
O2	0.0229 (7)	0.0313 (9)	0.0272 (9)	−0.0017 (7)	−0.0050 (6)	0.0068 (7)
O3	0.0184 (7)	0.0243 (7)	0.0114 (6)	0.0016 (6)	0.0005 (5)	0.0028 (5)
O4	0.0166 (6)	0.0244 (7)	0.0133 (6)	−0.0004 (6)	0.0038 (5)	−0.0033 (6)
O5	0.0166 (6)	0.0255 (7)	0.0149 (7)	0.0045 (6)	0.0004 (5)	0.0027 (6)
C1	0.0318 (12)	0.0426 (15)	0.0256 (12)	−0.0123 (11)	−0.0095 (9)	0.0101 (10)
C2	0.0455 (15)	0.0456 (15)	0.0113 (9)	−0.0270 (12)	0.0002 (10)	−0.0011 (10)
C3	0.0218 (9)	0.0340 (12)	0.0182 (10)	−0.0103 (10)	0.0068 (8)	−0.0077 (9)
C4	0.0156 (8)	0.0214 (9)	0.0142 (9)	−0.0018 (7)	0.0018 (7)	0.0023 (7)
C5	0.0161 (8)	0.0217 (9)	0.0126 (8)	−0.0029 (7)	0.0028 (7)	0.0009 (7)
C6	0.0154 (8)	0.0185 (9)	0.0175 (9)	0.0017 (7)	0.0017 (7)	−0.0001 (8)
C7	0.0126 (8)	0.0226 (10)	0.0149 (8)	0.0008 (7)	0.0017 (7)	−0.0017 (7)
C8	0.0142 (8)	0.0190 (9)	0.0134 (8)	−0.0022 (7)	0.0008 (6)	−0.0009 (7)
C9	0.0145 (7)	0.0187 (9)	0.0130 (8)	−0.0004 (7)	0.0001 (7)	−0.0006 (7)
C10	0.0162 (8)	0.0154 (8)	0.0112 (8)	−0.0023 (7)	−0.0006 (7)	−0.0001 (7)
C11	0.0141 (7)	0.0167 (8)	0.0127 (8)	−0.0017 (7)	0.0035 (6)	−0.0018 (7)
C12	0.0139 (8)	0.0160 (9)	0.0153 (9)	0.0003 (7)	−0.0017 (6)	−0.0016 (7)
C13	0.0164 (8)	0.0195 (9)	0.0107 (8)	−0.0022 (7)	0.0008 (7)	0.0002 (7)
C14	0.0185 (9)	0.0226 (10)	0.0157 (9)	0.0005 (8)	−0.0033 (7)	0.0026 (8)
C15	0.0196 (9)	0.0214 (10)	0.0262 (10)	−0.0027 (8)	0.0090 (8)	−0.0005 (9)
C16	0.0193 (9)	0.0240 (10)	0.0161 (9)	0.0014 (8)	−0.0025 (8)	0.0046 (8)

### Geometric parameters (Å, °)

**Table:**

O1—C5	1.224 (3)	C7—C8	1.461 (3)
O2—C1	1.353 (3)	C7—H7A	0.93 (3)
O2—C4	1.379 (3)	C8—C9	1.399 (3)
O3—C10	1.364 (2)	C8—C13	1.403 (3)
O3—C14	1.435 (2)	C9—C10	1.398 (3)
O4—C11	1.372 (2)	C9—H9A	0.93 (3)
O4—C15	1.438 (3)	C10—C11	1.400 (3)
O5—C12	1.366 (2)	C11—C12	1.407 (3)
O5—C16	1.437 (2)	C12—C13	1.387 (3)
C1—C2	1.332 (5)	C13—H13A	0.96 (3)
C1—H1A	1.03 (3)	C14—H14C	0.96 (3)
C2—C3	1.454 (4)	C14—H14B	0.99 (3)
C2—H2A	0.80 (5)	C14—H14A	0.93 (3)
C3—C4	1.357 (3)	C15—H15A	1.02 (5)
C3—H3A	0.76 (3)	C15—H15B	1.01 (3)
C4—C5	1.466 (3)	C15—H15C	0.96 (3)
C5—C6	1.477 (3)	C16—H16A	0.98 (3)
C6—C7	1.340 (3)	C16—H16B	0.97 (3)
C6—H6A	0.96 (3)	C16—H16C	1.00 (3)
			
C1—O2—C4	106.4 (2)	O3—C10—C9	124.33 (18)
C10—O3—C14	116.54 (16)	O3—C10—C11	115.57 (17)
C11—O4—C15	114.47 (16)	C9—C10—C11	120.10 (17)
C12—O5—C16	116.60 (16)	O4—C11—C10	119.52 (17)
C2—C1—O2	111.0 (2)	O4—C11—C12	120.68 (17)
C2—C1—H1A	137.1 (19)	C10—C11—C12	119.74 (17)
O2—C1—H1A	111.8 (19)	O5—C12—C13	124.26 (18)
C1—C2—C3	107.3 (2)	O5—C12—C11	115.50 (17)
C1—C2—H2A	135 (3)	C13—C12—C11	120.25 (17)
C3—C2—H2A	118 (3)	C12—C13—C8	119.85 (18)
C4—C3—C2	104.4 (2)	C12—C13—H13A	121.0 (15)
C4—C3—H3A	122 (3)	C8—C13—H13A	119.1 (15)
C2—C3—H3A	133 (3)	O3—C14—H14C	111.7 (17)
C3—C4—O2	110.8 (2)	O3—C14—H14B	110.3 (16)
C3—C4—C5	131.1 (2)	H14C—C14—H14B	112 (2)
O2—C4—C5	117.99 (17)	O3—C14—H14A	109.5 (18)
O1—C5—C4	119.79 (18)	H14C—C14—H14A	107 (3)
O1—C5—C6	123.80 (19)	H14B—C14—H14A	106 (2)
C4—C5—C6	116.36 (18)	O4—C15—H15A	109 (3)
C7—C6—C5	121.40 (19)	O4—C15—H15B	107.9 (19)
C7—C6—H6A	120.0 (17)	H15A—C15—H15B	108 (3)
C5—C6—H6A	118.1 (17)	O4—C15—H15C	106.8 (19)
C6—C7—C8	126.96 (19)	H15A—C15—H15C	116 (3)
C6—C7—H7A	118.4 (18)	H15B—C15—H15C	109 (3)
C8—C7—H7A	114.6 (18)	O5—C16—H16A	110.7 (17)
C9—C8—C13	120.28 (17)	O5—C16—H16B	105.3 (17)
C9—C8—C7	118.13 (18)	H16A—C16—H16B	109 (2)
C13—C8—C7	121.57 (17)	O5—C16—H16C	111.9 (18)
C10—C9—C8	119.77 (17)	H16A—C16—H16C	111 (2)
C10—C9—H9A	118.2 (18)	H16B—C16—H16C	109 (2)
C8—C9—H9A	122.0 (18)		
			
C4—O2—C1—C2	−0.1 (3)	C14—O3—C10—C11	179.43 (17)
O2—C1—C2—C3	0.1 (3)	C8—C9—C10—O3	−179.95 (18)
C1—C2—C3—C4	−0.1 (3)	C8—C9—C10—C11	0.1 (3)
C2—C3—C4—O2	0.0 (2)	C15—O4—C11—C10	104.9 (2)
C2—C3—C4—C5	−175.9 (2)	C15—O4—C11—C12	−77.7 (2)
C1—O2—C4—C3	0.1 (3)	O3—C10—C11—O4	−2.9 (3)
C1—O2—C4—C5	176.5 (2)	C9—C10—C11—O4	177.11 (18)
C3—C4—C5—O1	−4.4 (4)	O3—C10—C11—C12	179.71 (18)
O2—C4—C5—O1	179.97 (19)	C9—C10—C11—C12	−0.3 (3)
C3—C4—C5—C6	172.9 (2)	C16—O5—C12—C13	1.4 (3)
O2—C4—C5—C6	−2.7 (3)	C16—O5—C12—C11	−178.70 (17)
O1—C5—C6—C7	8.0 (3)	O4—C11—C12—O5	3.4 (3)
C4—C5—C6—C7	−169.2 (2)	C10—C11—C12—O5	−179.21 (17)
C5—C6—C7—C8	174.83 (19)	O4—C11—C12—C13	−176.68 (18)
C6—C7—C8—C9	173.3 (2)	C10—C11—C12—C13	0.7 (3)
C6—C7—C8—C13	−8.3 (3)	O5—C12—C13—C8	179.05 (18)
C13—C8—C9—C10	−0.2 (3)	C11—C12—C13—C8	−0.8 (3)
C7—C8—C9—C10	178.14 (18)	C9—C8—C13—C12	0.6 (3)
C14—O3—C10—C9	−0.6 (3)	C7—C8—C13—C12	−177.69 (19)

### Hydrogen-bond geometry (Å, °)

**Table:**

*D*—H···*A*	*D*—H	H···*A*	*D*···*A*	*D*—H···*A*
C14—H14B···O1^i^	0.99 (3)	2.54 (3)	3.503 (3)	166 (2)
C15—H15B···O4^ii^	1.01 (3)	2.36 (3)	3.337 (3)	162 (2)

Symmetry codes: (i) −*x*+1/2, *y*−1/2, *z*−1/2; (ii) *x*, *y*+1, *z*.
